# Sugar Promotes Feeding in Flies via the Serine Protease Homolog *scarface*

**DOI:** 10.1016/j.celrep.2018.08.059

**Published:** 2018-09-18

**Authors:** Naveen Prasad, Korneel Hens

**Affiliations:** 1 Centre for Neural Circuits and Behaviour, The University of Oxford, Tinsley Building, Mansfield Road, Oxford OX1 3SR, UK

**Keywords:** feeding behavior, macronutrient, Scarface, hedonic control, *Drosophila*, carbohydrates

## Abstract

A balanced diet of macronutrients is critical for animal health. A lack of specific elements can have profound effects on behavior, reproduction, and lifespan. Here, we used *Drosophila* to understand how the brain responds to carbohydrate deprivation. We found that serine protease homologs (SPHs) are enriched among genes that are transcriptionally regulated in flies deprived of carbohydrates. Stimulation of neurons expressing one of these SPHs, Scarface (Scaf), or overexpression of *scaf* positively regulates feeding on nutritious sugars, whereas inhibition of these neurons or knockdown of *scaf* reduces feeding. This modulation of food intake occurs only in sated flies while hunger-induced feeding is unaffected. Furthermore, *scaf* expression correlates with the presence of sugar in the food. As Scaf and Scaf neurons promote feeding independent of the hunger state, and the levels of *scaf* are positively regulated by the presence of sugar, we conclude that *scaf* mediates the hedonic control of feeding.

## Highlights

• The fly brain responds to specific macronutrients via distinct signaling pathways
• Serine protease homologs act as neuromodulators under sugar deprivation
• Sugar is both necessary and sufficient to maintain expression levels of *scarface*
• Scarface and Scarface neurons mediate hedonic control of feeding in flies

## Graphical Abstract

**Figure:**
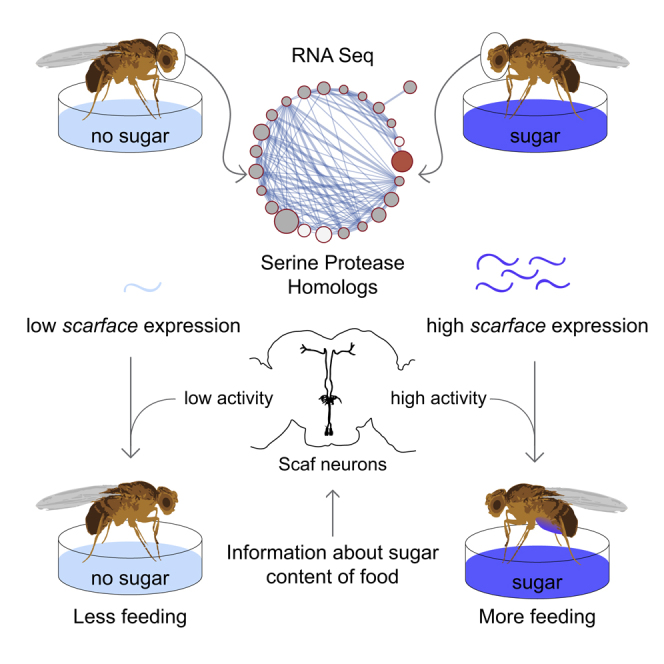

## Teaser

Regulation of feeding on nutritious food is critical for survival. Using an unbiased, transcriptional-based approach, Prasad and Hens identify serine protease homologs as neuromodulators in the fly brain. The authors elucidate the role of *scarface* and neurons labeled by Scarface in promoting feeding on nutritious food without evoking hunger.

## Introduction

Nutrient homeostasis is a basic biological process that involves adjusting feeding behavior and post-digestive physiology to balance food intake with energy expenditure. The CNS is a key regulator of nutritional homeostasis in species ranging from worms to humans. The CNS maintains homeostasis by coordinating food intake and utilization in response to internal and external cues using different neuronal circuits and peptide hormones (Morton et al., 2006, Peters et al., 2007, Porte et al., 2005). Disruption of this homeostatic balance can lead to a number of diet-related disorders such as obesity and type 2 diabetes.

The role of macronutrient balance in the management of dietary disorders has been extensively studied (Ebbeling et al., 2012, Goss et al., 2013, Lee, 2015, Solon-Biet et al., 2014, Te Morenga and Mann, 2012). Changes in carbohydrate levels affect sleep, locomotion, longevity, and immunity in *Drosophila* (Catterson et al., 2010, Galenza et al., 2016, Morris et al., 2012, Na et al., 2013). Regulation of gene expression is an important mechanism that helps maintaining nutrient homeostasis in the CNS (Desvergne et al., 2006). Mice fed a high-fat diet modulate expression of genes controlling dopamine availability in the hypothalamus (Lee et al., 2010), while CREB-regulated transcription co-activator 2 links hypothalamic glucose sensing with expression of the *insulin receptor substrate 2* gene (Lerner et al., 2009). In the *Drosophila* brain, the expression of two insulin genes is reduced upon starvation (Ikeya et al., 2002). In addition, cessation of larval feeding activity coincides with downregulation of neuropeptide F expression in the brain (Wu et al., 2003). Many peptidergic neurons also contain fast-acting neurotransmitters (van den Pol, 2012) and individual neurons can express multiple neuropeptides (Everitt et al., 1986). Transcriptional regulation provides a mechanism to maintain or modulate the ratio at which such co-expressed peptides are released by regulating their expression. Furthermore, it is likely that gene families other than neuropeptides are transcriptionally regulated to maintain nutrient homeostasis, but these remain less well characterized. It is therefore important to obtain a systems-level view of the transcriptional changes that occur upon changes in the diet.

The fruit fly *Drosophila melanogaster* has been successfully used as a model to study diet-related disorders (Owusu-Ansah and Perrimon, 2014) and identify genes in mammals that are involved in obesity and glucose metabolism (Buchmann et al., 2007, Pospisilik et al., 2010, Ugrankar et al., 2015). Here, we mapped transcriptional changes taking place in the *Drosophila* brain to investigate the mechanisms by which nutritional homeostasis is maintained under conditions of carbohydrate starvation. Since sugar is a component of food, it might be expected that the genes that are transcriptionally regulated upon sugar starvation are a subset of those that are altered under complete starvation. We indeed found that the response to sugar starvation contained some features of the response to complete starvation. However, we also observed a large sugar-specific response. Further analysis of the data revealed a family of proteins, serine protease homologs (SPHs), that might play an important role in the maintenance of homeostasis under changing levels of sugar in the food. We studied the role of neurons that express one of these SPHs, Scarface (Scaf), in the modulation of fly behavior. We demonstrate that *scaf* as well as Scaf neurons promote feeding independent of the hunger state. Instead, *scaf* promotes feeding in response to the presence of sugar in the food, therefore providing a mechanism for the regulation of hedonic feeding.

## Results

### The Transcriptional Response of the Fly Brain to Sugar Starvation Is Distinct from the Response to Complete Starvation

Previous studies have explored the transcriptional changes in response to complete starvation in whole adult males (Grönke et al., 2005, Moskalev et al., 2015) and heads of adult female flies (Chatterjee et al., 2014, Fujikawa et al., 2009). Other studies have compared the transcriptional effects of complete starvation to feeding on a diet consisting only of sugar in whole larvae (Zinke et al., 2002) and adults (Bauer et al., 2006). However, gene expression changes in the entire body or head may obscure gene expression changes specific to the brain. Furthermore, genome-wide mapping of differential gene expression in response to deprivation specifically of one macronutrient has not been performed in *Drosophila*. We used a holidic medium (Piper et al., 2014) to specifically deprive flies of carbohydrates and carried out transcriptional analyses in brains. Sugar (sucrose) is the only source of carbohydrates in the holidic medium, and hence, we will be referring to carbohydrate starvation as sugar starvation.

**Figure 1:**
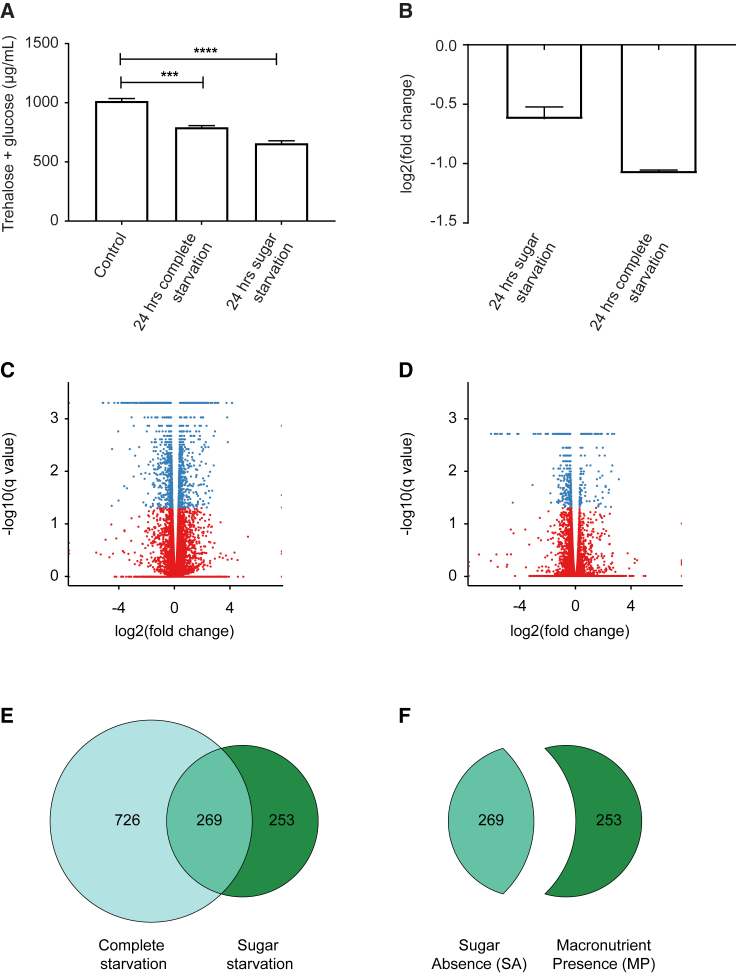
Transcriptional Response of the Fly Brain to Sugar Starvation Is Distinct from the Response to Complete Starvation (A) Combined levels of circulating trehalose and glucose are significantly decreased after 24 hr of sugar or complete starvation (n = 4). Error bars represent SEM. ^∗∗∗^p ≤ 0.001, ^∗∗∗∗^p ≤ 0.0001. (B) Transcript levels of *ilp2* and *ilp5* in the fly brain after 24 hr of sugar and complete starvation as measured by qRT-PCR (n = 4). Error bars represent SD. (C and D) Volcano plots showing differentially expressed genes under conditions of complete starvation (C) and sugar starvation (D), comparing fold change values to their statistical significance levels. Blue dots represent differentially expressed genes with a q value ≤0.05 (significant change), while the red dots indicate those with q values >0.05. (E) Venn diagram showing overlap of the differentially expressed genes after complete and sugar starvation. (F) Two subsets of the sugar starvation dataset divided based on their overlap with the complete starvation dataset. See also Figure S1.

We first tested whether 24 hr of sugar and complete starvation is sufficient to elicit a homeostatic response in flies by measuring the combined levels of circulating trehalose and glucose. These levels were significantly reduced following sugar and complete starvation as compared to *ad libitum* fed controls (Figure 1A). Interestingly, the reduction in circulating sugars was more pronounced in sugar-starved flies. It is possible that complete starvation leads to a stronger release of glucose from storage through glycogenolysis and trehaloneogenesis, thus buffering the reduction of circulating glucose and trehalose levels. Furthermore, sugar-starved flies may derive additional energy from the ketogenic degradation of the amino acids present in the food, resulting in lower circulating glucose and trehalose levels as compared to the complete starvation condition. We also measured *insulin-like peptide 5* (*ilp5*) mRNA levels under these starvation regimes. The *ilp5* transcript level was reduced upon 24 hr of sugar as well as complete starvation, consistent with a previous study (Birse et al., 2011; Figure 1B). We therefore used a 24-hr starvation regime for subsequent transcriptome analysis.

To study tissue-specific responses to dietary challenge, we performed RNA sequencing (RNA-seq) on brains from adult male flies that were starved for sugar, starved completely, or fed *ad libitum* (control). We identified 995 genes that were differentially expressed in the brain under complete starvation conditions (Figure 1C; Table S1) and 522 genes that were differentially expressed in the brain upon sugar starvation (Figure 1D; Table S2), as compared to *ad libitum* fed controls. We found that 51% of the genes that were differentially expressed under sugar starvation were also differentially expressed under complete starvation (Figure 1E). Since sugar is absent in both sugar and complete starvation regimes, one might expect that the sugar starvation dataset should be a subset of the complete starvation dataset. However, our data show that when flies are provided with food that has all nutrients except sugar, the brain responds to this challenge in a manner that is distinct from the response when deprived of all nutrients.

We validated our results by qRT-PCR on a fifth independent biological replicate for both datasets for randomly selected genes covering a wide range of differential expression levels (Figure S1) and found good correlation with our RNA-seq data.

### The Transcriptional Response to the Absence of Sugar Is Mediated through the Enzyme-Linked Receptor Protein Signaling Pathway

**Figure 2:**
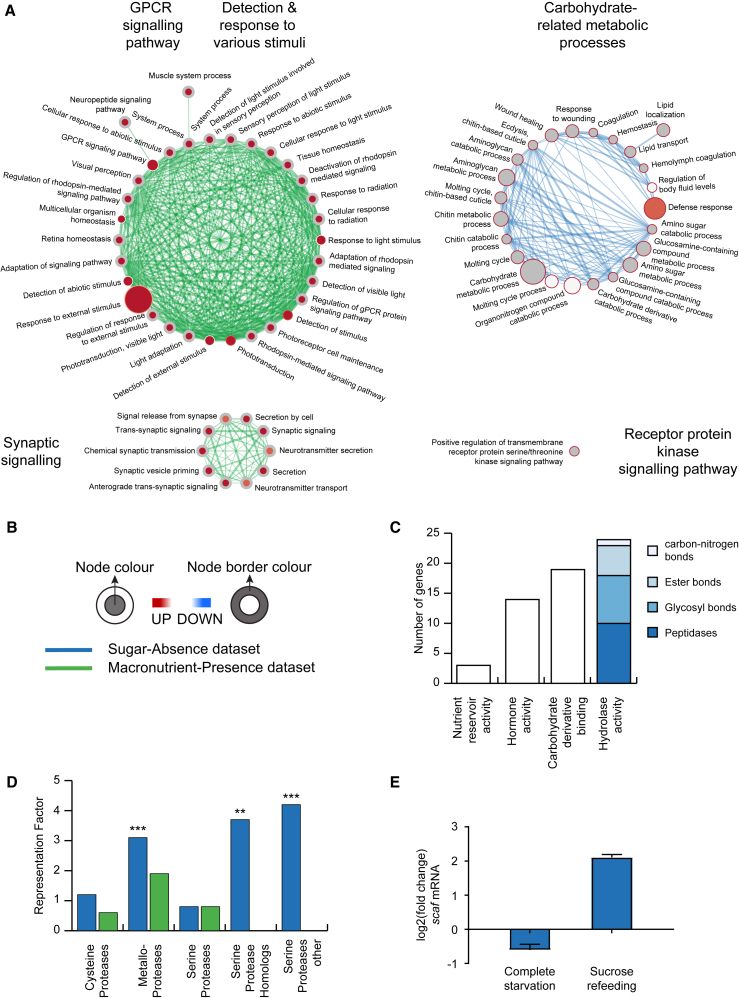
SPHs Are Important Mediators of the Maintenance of Energy Homeostasis (A) Comparison of the enrichment of biological processes associated with sugar-absence (SA) and macronutrient-presence (MP) datasets visualized by the Cytoscape enrichment app. The node (inner circle) size corresponds to the number of genes in the MP dataset, while the node border (outer circle) size corresponds to those in the SA dataset within the given biological process. The color of the node and border corresponds to the significance of the enrichment. Edge size (lines connecting the nodes) corresponds to the number of genes that overlap between the two connected biological processes. Green edges correspond to the MP dataset, and blue corresponds to SA dataset. The network map was manually curated removing general and uninformative sub-networks. The full network map can be seen in Data S1. (B) Legends for (A) and (D). (C) Molecular functions enriched within the secreted proteins of the SA dataset. The largest parent GO term (hydrolase activity) is further divided into its constituent child terms. (D) Representation factors of different protease families in the SA (blue) and MP (green) datasets. ^∗∗^p ≤ 0.01, ^∗∗∗^p ≤ 0.001. (E) Relative transcript levels of *scaf* in heads of flies under different feeding conditions. Flies were starved of all nutrients for 24 hr and subsequently starved for an additional 3 hr (complete starvation) or fed with a 200 mM sucrose solution (sucrose refeeding). Scaf expression levels in the fly head were measured by qRT-PCR and compared to *scaf* expression levels of flies that were fully fed for 27 hr (controls) (n = 3). Error bars represent SD. See also Figure S2.

To better understand the molecular events that mediate the brain’s response to the absence of sugar, we focused on the genes that were differentially expressed under sugar starvation. We divided these genes into two subgroups: those that are differentially expressed under both sugar and complete starvation regimes and those that showed differential expression only following sugar deprivation (Figure 1F). The genes in the first group mediate the response of the brain specifically to the absence of sugar regardless of the presence or absence of other macronutrients. Hence, we will call them the sugar-absence (SA) genes. The genes in the second group mediate the response of the brain to the presence of macronutrients other than sugar in the food. We will call those genes the macronutrient-presence (MP) genes. Biological processes related to sugar or carbohydrate metabolism and processes linked to energy utilization such as sleep and post-mating behavior are enriched specifically in the SA dataset (Figures 2A and 2B; Data S1). Within the MP dataset, processes related to detection and response to external stimuli and synaptic signaling, long-term memory, and aging are enriched. Interestingly, the MP and SA responses appear to be mediated through different signaling systems (Figures 2A and 2B; Data S1). The SA dataset is enriched for genes that are associated with the enzyme-linked receptor protein signaling pathway, such as *insulin-like peptide 2* (*ilp2*), *ilp5*, and *secreted decoy of InR and ecdysone-inducible gene L2*, which are well known to regulate energy homeostasis. The MP dataset is enriched for genes involved in the G-protein-coupled receptor (GPCR) signaling pathway, including several genes that have been implicated in the regulation of feeding behavior and nutrient preference, such as *Allatostatin Receptor 1*, *neuropeptide F*, and *bride of sevenless*.

### Genes Responding to the Absence of Sugar Encode Mainly Secreted Proteins

We next categorized the genes in the SA and MP datasets based on the predicted subcellular localization of the proteins they encode (Figures S2A and S2B; Data S1). The SA dataset is enriched for gene products that localize to the extracellular region (74 out of 269 genes, p < 1 × 10^−22^), indicating that many of these proteins are secreted. The MP dataset is enriched for gene ontology (GO) terms such as “Synapse” (p < 0.005) and “Neuron part” (p < 0.05) that indicate proteins that localize to synaptic vesicles and parts of the neuron (21 out of 253 genes). It appears that to meet the energy challenges posed by lack of sugar, the brain modulates expression of secreted proteins, which can have effects on the physiology of the entire body, potentially eliciting responses in tissues that have little neural innervation, such as the fat body and the oenocytes. In contrast, the sugar-starvation-specific gene products are mainly localized in neurons, suggesting that the additional response mounted by the brain under sugar starvation regime is largely effected by innervated tissues.

### SPHs Are Important Mediators of the Maintenance of Energy Homeostasis

Comparing the transcriptome changes in completely starved and sugar-starved flies provides us with a repertoire of genes that change expression upon carbohydrate deprivation. To show that this resource can be mined to understand the molecular mechanisms of the responses of the brain to carbohydrate deficiency, we studied a gene from the SA dataset with no known role in the regulation of nutrient homeostasis. As the genes that mediate the brain’s response to the absence of sugar are significantly enriched for genes that code for secreted proteins, we determined the GO enrichment of the molecular function of these secreted proteins. Proteins with hydrolase activity were the largest enriched family (24 out of 74 genes; p < 0.001) (Figure 2C). Within that group, proteins with peptidase activity were the most abundant (10 out of 24 genes). We therefore classified all peptidases in the SA dataset into different peptidase families using the MEROPS peptidase database (Rawlings et al., 2016) and calculated the representation of these families in both the SA and MP datasets. SPHs and serine protease (SP)-other were exclusively enriched in the SA dataset (Figures 2B and 2D). SPHs are similar in amino acid sequence to S1 family SPs but lack amidase activity, because one or more of the catalytic residues are missing. It has been suggested that catalytically inactive enzymes can adopt novel roles in regulatory processes (Pils and Schultz, 2004). This raises the intriguing possibility that SPHs play a neuromodulatory role in the brain.

From the list of SPHs, we chose to study the role of Scaf in nutrient homeostasis. Scaf is secreted (Ross et al., 2003) and a target of an enzyme-linked receptor protein signaling pathway, namely the JNK signaling pathway (Rousset et al., 2010). Scaf expression is downregulated in both complete and sugar starvation, suggesting that sugar is necessary to maintain the levels of *scaf*. To test whether sugar alone is also sufficient to maintain the levels of *scaf*, we starved flies of all nutrients for 24 hr. Subsequently, these flies were starved for an additional 3 hr (complete starvation) or fed with a 200 mM sucrose solution (sucrose refeeding). *Scaf* expression levels in the fly head were measured by qRT-PCR and compared to *scaf* expression levels of flies that were fully fed for 27 hr (controls). In starved flies, *scaf* the RNA level dropped to almost half of its original value, in line with our RNA-seq data. In the flies fed with sucrose, levels of *scaf* were strongly enhanced (Figure 2E). These experiments show that sugar in the food is both necessary and sufficient to maintain transcript levels of *scaf*. To test whether the enhanced expression level of *scaf* upon refeeding is specific to sucrose, we repeated the experiment with two nutritive (D-fructose and D-glucose) and three non-nutritive (L-glucose, sucralose, and D-arabinose) sugars. We also performed the experiments with sorbitol, a sugar alcohol, and amino acids (Figure S2C). Refeeding nutritive sugars as well as sorbitol and amino acids results in *scaf* expression at or above pre-starvation levels. Nonnutritive sugars fail to raise *scaf* expression levels to or above pre-starvation levels. These results may indicate that *scaf* expression responds to the nutritional quality of the food rather than to a specific sugar, although the effect of sucrose refeeding on scaf expression is at least 2-fold higher than with any of the other nutrients we tested.

### Scaf Is Expressed in the Subesophageal Zone of the Brain and the Ventral Nerve Cord

**Figure 3:**
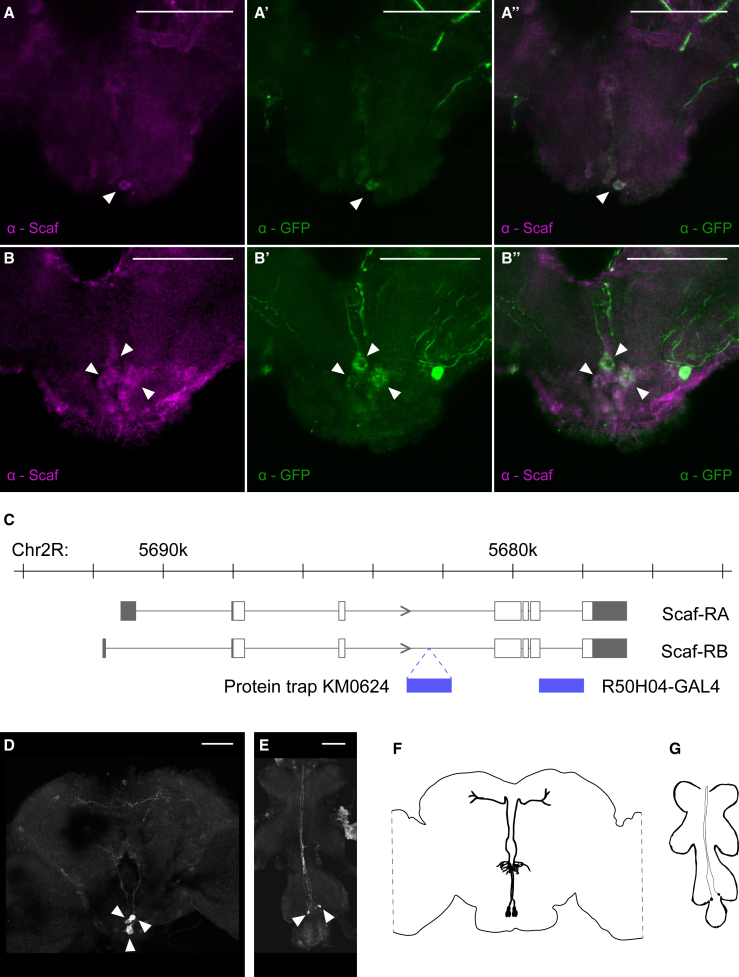
Scaf Is Expressed in the Subesophageal Zone of the Brain and the Ventral Nerve Cord (A–B′′) The SEZ of adult brains from R50H04-GAL4; UAS-6XGFP flies double stained using anti-GFP (green) and anti-Scaf (magenta) antibodies demonstrating that Scaf is expressed in R50H04 neurons. Arrowheads mark the location of cell bodies expressing Scaf and GFP. (A)–(A′′) and (B)–(B′′) represent anterior and posterior focal planes, respectively. (C) Gene structure of *scaf*. Black rectangles indicate UTRs, and white rectangles indicate coding exons. The blue bars indicate the insertion site of the protein trap and the intronic region used to generate the R50H04-GAL4 line. (D and E) Adult fly brain (D) and VNC (E) from R50H04-GAL4; UAS-mCD8::GFP flies stained with an anti-GFP antibody (white) showing the cell bodies and neuronal processes of Scaf neurons. (F and G) Schematic representation of the cell bodies and projections of Scaf neurons in brain (F) and VNC (G). Scale bars, 50 μm. See also Figures S3–S5 and Videos S1 and S2.

We determined the distribution of Scaf protein in the brain using anti-Scaf antibodies (Rousset et al., 2010). We observed Scaf immunostaining in a limited number of neurons whose cell bodies are located in the SEZ (Figures 3A, 3B, S3A, and S3B). Next, we looked for regulatory sequences within the *scaf* locus that drive the observed expression pattern. The R50H04-GAL4 line contains an intronic region of the *scaf* gene upstream of the GAL4 coding sequence (Jenett et al., 2012) (Figure 3C). Double labeling brains from R50H04-GAL4; UAS-6XGFP flies with anti-Scaf and anti-GFP confirmed that R50H04-GAL4 labels Scaf-expressing neurons (Figures 3A–3B′′ and S3A–S3B′′). We also analyzed the expression of a protein trap line that has a GFP fusion construct inserted into the endogenous *scaf* genomic locus (PBac(EGFP-IV)scaf^KM0624^) (Figure 3C). Double labeling brains from R50H04-GAL4/PBac(EGFP-IV)scaf^KM0624^; UAS-mCherry flies further confirms that R50H04-GAL4 labels Scaf-expressing neurons (Figures S3C–S3C′′). Consequently, we will refer to R50H04-GAL4-labeled cells as Scaf neurons.

We used R50H04-GAL4 to describe the anatomy of Scaf neurons. R50H04 labels neurons in the anterior part of the SEZ and in the posterior part. R50H04 also labels two neurons in the VNC (Figure 3D–3G and S4). The projections of these neurons are difficult to appreciate in 2D due to the weakness of the GAL4 driver. We have therefore made a 3D reconstruction of the brain in Figure S4 at higher laser intensity (Videos S1 and S2), showing that the cell bodies in the SEZ send projections around the esophageal foramen in a glomerular-like organization and to the protocerebrum. To determine the polarity of Scaf neurons, we used R50H04-GAL4 to drive the expression of the presynaptic GFP-dSyd (Owald et al., 2010) and the postsynaptic DenMark-RFP (Nicolaï et al., 2010) markers (Figure S5). DenMark-labeled processes are present around the foramen and in the SEZ. DSyd-marked processes extend into the protocerebrum. Although we do not know the identity of the neurons in the SEZ or in the protocerebrum that make synaptic connections with Scaf neurons, Scaf neuron polarity suggests that they carry information from the SEZ to higher centers of the brain.

Video S1. 3D Reconstruction of Confocal Image Stack from R50H04-GAL4; UAS-GFP Flies, Related to Figure 3

Video S2. 3D Reconstruction of Confocal Image Stack from R50H04-GAL4; UAS-GFP Flies, Related to Figure 3

### Scaf Promotes Feeding in Response to the Sugar Levels in the Food

The downregulation of *scaf* expression upon sugar deprivation and its expression in the SEZ suggests that Scaf has a role in the regulation of feeding. We therefore tested the effect of overexpression and knockdown of *scaf* on feeding using two feeding assays: a dye-feeding assay (Burke and Waddell, 2011) and the capillary feeder (CAFÉ) assay (Ja et al., 2007). The CAFÉ assay can be used to accurately measure feeding over longer periods but requires the fly to climb and feed upside down. The dye assay can only be used during the initial phase of feeding before excretion of the dye (Wong et al., 2008) but does not require the fly to climb in order to feed.

**Figure 4:**
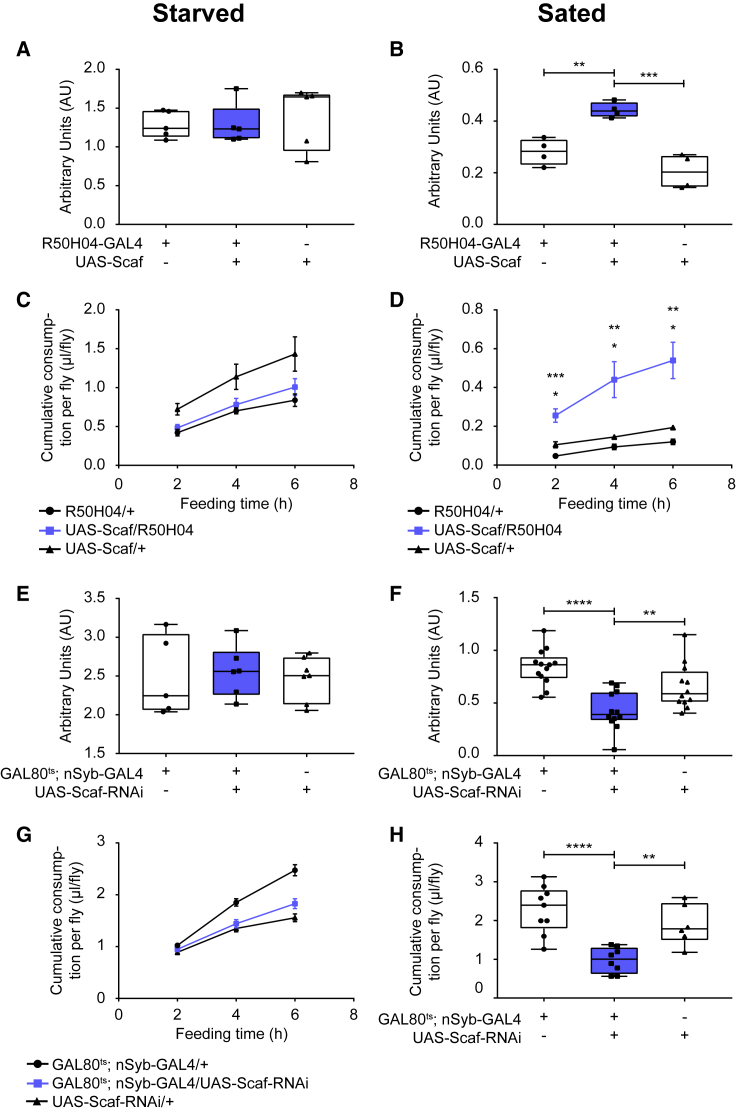
Scaf Promotes Feeding of Sucrose in Flies (A and B) Effect of *scaf* overexpression on feeding over a short period. Scaf overexpression was driven by R50H04-GAL4, and feeding was quantified using the dye assay at 25°C for 15 min. (A) Overexpression of *scaf* does not change starvation-induced feeding in flies upon 16 hr of starvation (n = 5–6). (B) Overexpression of *scaf* results in enhancement of feeding in sated flies (n = 4–5). (C and D) Effect of *scaf* overexpression on feeding over longer periods. Scaf overexpression was driven by R50H04-GAL4, and feeding was quantified at 25°C for 2, 4, and 6 hr using the CAFÉ assay. (C) Overexpression of *scaf* does not change starvation-induced feeding in flies upon 16 hr of starvation (n = 4). (D) Overexpression of *scaf* results in enhancement of feeding in sated flies (n = 4–6). (E and F) Effect of *scaf* knockdown on feeding over a short period. Scaf RNAi was driven by *n-syb*-GAL4 in adults upon tub-GAL80^ts^ de-repression by transferring the flies to 30°C for 6 days. Feeding was quantified using the dye assay at 30°C for 15 min (E) and 30 min (F). (E) Knockdown of *scaf* does not change starvation-induced feeding in flies upon 16 hr of starvation (n = 6–7). (F) Knockdown of *scaf* results in decreased feeding in sated flies (n = 12–13). (G and H) Effect of *scaf* knockdown on cumulative feeding. Scaf RNAi was driven by *n-syb*-GAL4 in adults upon tub-GAL80^ts^ de-repression by transferring the flies to 30°C for 6 days. (G) Feeding was quantified at 30°C for 2, 4, or 6 hr after 16 hr of starvation using the CAFÉ assay. Knockdown of *scaf* does not change starvation-induced feeding in flies (n = 9–10). (H) Feeding was quantified at 30°C for 24 hr in sated flies using the CAFÉ assay. Knockdown of *scaf* results in decreased feeding in sated flies (n = 7–10). (B, D, F, and H) ^∗∗^p ≤ 0.01, ^∗∗∗^p ≤ 0.001, ^∗∗∗∗^p ≤ 0.0001. Whiskers in all box plots go from the minimum to the maximum value, while the box extends from 25th to 75th percentile. All values of individual groups of flies are shown as dots and the line in the box is plotted at the median. Line graphs represent the mean. Error bars represent SEM. See also Figure S6.

We first overexpressed *scaf* in Scaf neurons using R50H04-GAL4 and measured feeding post-starvation using both assays (Figures 4A and 4C). We quantified feeding for 15 min using the dye assay and for 2, 4, and 6 hr using the CAFÉ assay. We did not observe any change in feeding upon *scaf* overexpression. However, overexpression of *scaf* caused increased feeding in sated flies (Figures 4B and 4D). The data suggest that *scaf* promotes feeding, although it does not increase food intake in hungry flies. It is possible that *scaf* promotes hunger signals or inhibits satiety signals, which would cause increased feeding in sated flies. In starved flies, the motivation to feed is already high. Consequently, additional hunger signals may not cause observable changes in feeding.

To test whether the increased feeding upon *scaf* overexpression in sated flies is specific to sucrose as the substrate for caloric repletion, we repeated the experiment with fructose as substrate under similar conditions. We did not observe a difference in feeding between control and overexpression flies (Figures S6A and S6B), both in starved and sated conditions, when feeding was quantified for 15 min. We did notice that feeding was generally lower on fructose than on sucrose in these conditions. We therefore increased the feeding time to 30 min, after which a difference in feeding between control and overexpression flies started to appear in the sated state, but not in the starved state (Figures S6C and S6D). This phenotype was confirmed when feeding was measured over longer periods in the CAFÉ assay (Figures S6E and S6F). Consequently, the effects of *scaf* overexpression on feeding seems to be directed toward other nutritious sugars besides sucrose as well.

Next, we checked if knockdown of *scaf* has opposite effects on feeding. To downregulate *scaf*, we used the strong pan-neuronal driver *Synaptobrevin-*GAL4 (*n-Syb*-GAL4) to drive *scaf*-RNAi to ensure sufficient knockdown of *scaf*. We used *tub*P-GAL80^ts^ to drive the expression of the transgene specifically in adults. We quantified feeding using the dye assay for 15 min and for 2, 4, and 6 hr using the CAFÉ assay. Downregulation of *scaf* did not change starvation-induced feeding (Figures 4E and 4G). These data argue against a role of *scaf* in modulating hunger or satiety signals, as decreasing hunger signals or increasing satiety signals would result in decreased feeding in starved flies. It is therefore unlikely that *scaf* is part of the genetic mechanisms that mediate starvation-induced feeding. The data also suggest that *scaf* does not directly affect the general feeding motor program, as *scaf* knockdown does not inhibit starvation-induced feeding.

As overexpression of *scaf* results in enhanced feeding in sated state, it is likely that *scaf* promotes the continuation of feeding. To test this hypothesis, we knocked down *scaf* and checked feeding in sated flies for 30 min using the dye assay (Figure 4F) and for 24 hr using the CAFÉ assay (Figure 4H). Knockdown of *scaf* resulted in a significant reduction in feeding using both assays. The data show that although *scaf* does not affect starvation-induced feeding, it modifies continuation of feeding in flies. Combined with our data showing that sugar in the food is both necessary and sufficient to maintain levels of *scaf* (Figure 2E), we conclude that the presence of sugar in food can act as a signal to promote feeding by positively regulating *scaf* levels. As overexpression of *scaf* also leads to enhanced feeding of fructose under sated conditions, it is likely that other nutritive sugars have a similar impact on *scaf* and subsequently on feeding.

### Scaf Neurons Promote Feeding in Flies

**Figure 5:**
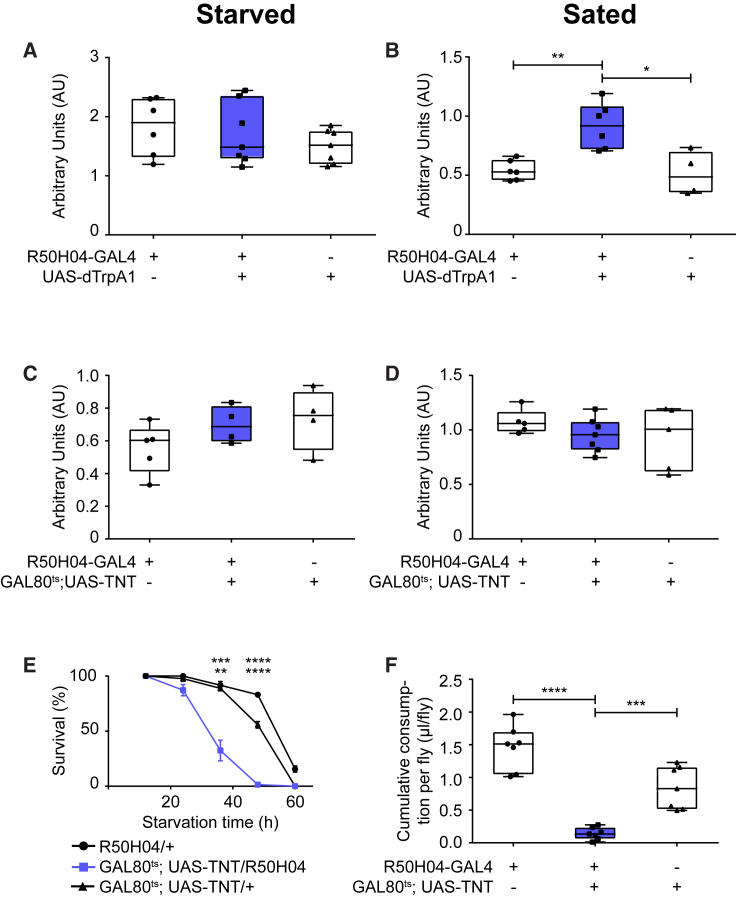
Scaf Neurons Promote Feeding of Sucrose in Flies (A and B) Effect of Scaf neuron activation on feeding. dTrpA1 expression was driven by R50H04-GAL4 to activate Scaf neurons for 1 hr by shifting the flies from 21°C to 31°C. Feeding was subsequently quantified in flies using the dye assay for 10 min at 31°C. (A) Activation of Scaf neurons does not change post-starvation feeding upon 16 hr of starvation (n = 6–7). (B) Activation of Scaf neurons results in increased feeding in sated flies (n = 4–7). (C and D) Effect of Scaf neuron silencing on feeding. Scaf neurons were silenced by de-repressing *tub*-GAL80^ts^ at 31°C for 48 hr, resulting in the expression of TNT driven by R50H04-GAL4. (C) Silencing Scaf neurons does not inhibit starvation-induced feeding in flies. Flies were starved for 16 hr at 25°C, and feeding was quantified using a dye assay for 15 min at 25°C (n = 5). (D) Silencing Scaf neurons does not show measurable difference in feeding in sated flies. The flies were recovered at 25°C, and feeding was quantified using the dye assay for 30 min at 25°C (n = 5–7). (E) Silencing of Scaf neurons results in decreased starvation resistance in adult flies. Scaf neurons were silenced by de-repressing tub-GAL80^ts^ by transferring the flies to 31°C for 48 hr, resulting in the expression of TNT driven by R50H04-GAL4. The flies were then transferred to starvation medium at 25°C, and lethality was calculated by counting the number of dead flies at different intervals (n = 4–6). (F) Silencing of Scaf neurons results in decreased feeding over longer periods. Scaf neurons were silenced by de-repressing tub-GAL80^ts^ by transferring the flies to 31°C for 48 hr, resulting in the expression of TNT driven by R50H04-GAL4. Feeding was quantified at 25°C over a 24-hr period using the CAFÉ assay (n = 7). (B, E, and F) ^∗^p ≤ 0.05, ^∗∗^p ≤ 0.01, ^∗∗∗^p ≤ 0.001, ^∗∗∗∗^p ≤ 0.0001. Whiskers in all box plots go from the minimum to the maximum value, while the box extends from 24th to 75th percentile. All values of individual groups of flies are shown as dots and the line in the box is plotted at the median. Line graphs represent the mean with error bars representing SEM. See also Figure S6.

As *scaf* positively regulates feeding and is expressed in R50H04 neurons, we tested whether Scaf neuron activity also regulates feeding. We activated Scaf neurons by ectopically expressing the heat-activated *Drosophila* transient receptor potential (dTrpA1) channel (Hamada et al., 2008) using R50H04-GAL4 and checked the effect on feeding for 10 min using the dye-feeding assay. Similar to *scaf* overexpression, activation of Scaf neurons did not modulate feeding in starved flies (Figure 5A) but increased feeding in sated flies (Figure 5B). We also tested the effect of activating Scaf neurons on feeding on fructose and observed a similar phenotype (Figures S6G and S6H).

Next, we silenced Scaf neurons by ectopic expression of tetanus toxin light chain (TNT) (Sweeney et al., 1995) using R50H04-GAL4. We also used *tub*P-GAL80^ts^ to suppress TNT expression during development. We quantified feeding in starved flies for 15 min using the dye assay. Silencing of Scaf neurons, like *scaf* knockdown, did not affect starvation-induced feeding (Figure 5C).

To test whether silencing of Scaf neurons leads to decreased feeding under *ad libitum* conditions, we silenced Scaf neurons and quantified feeding for 30 min in sated flies using the dye assay. In contrast to our findings with scaf knockdown, we did not observe any differences in feeding under these conditions (Figure 5D). Under sated conditions, parental control flies eat very little. It is possible that a decrease in feeding in the experimental flies would not be noticeable over a short period. The fact that we do see a difference in feeding upon *scaf* knockdown may be due to small differences in experimental design (see STAR Methods). Alternatively, as Scaf is a secreted protein, it may have cell-non-autonomous effects.

Quantifying feeding over a 24-hr period upon silencing Scaf neurons in sated flies did show a significant reduction in feeding (Figure 5F), suggesting that Scaf neurons, like *scaf*, regulate the continuation of feeding. It is possible, however, that silencing the Scaf neurons results in a reduced ability of the fly to climb and feed from the capillary, causing the reduction in feeding. To test this, we silenced Scaf neurons for 48 hr on regular food. Under these conditions, flies do not have to climb to feed. If silencing of Scaf neurons leads to decreased feeding under *ad libitum* conditions, then this would result in lower energy reserves and decreased starvation resistance. We indeed found that the flies in which Scaf neurons were silenced showed decreased starvation resistance (Figure 5E). Overall, our data show that similar to *scaf*, activity of Scaf neurons is required to promote feeding in flies under *ad libitum* conditions.

### Scaf Promotes Feeding Specifically on Nutritious Sugar Independent of the Hunger State

**Figure 6:**
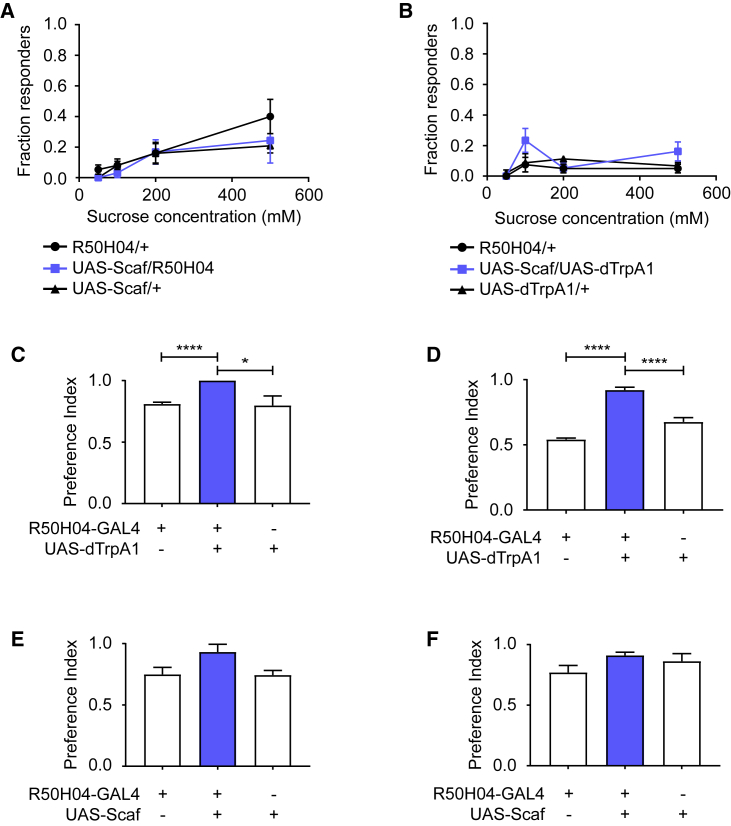
*Scaf* and Scaf Neurons Promote Feeding on Nutritious Sugar Independent of the State of the Fly (A) Overexpression of *scaf* does not result in enhanced PER at varying concentrations of sucrose (n = 4). (B) TrpA1-mediated activation of Scaf neurons does not lead to increased PER in sated flies at varying concentrations of sucrose (n = 4). Scaf neurons were activated by expressing dTrpA1 using R50H04-GAL4 by shifting the flies to 31°C for 1 hr. (C and D) Activation of Scaf neurons does not alter feeding preference. Scaf neurons were activated by expressing dTrpA1 using R50H04-GAL4 in sated flies. Preference was tested at 31°C under darkness for 2 hr. ^∗^p ≤ 0.05, ^∗∗∗∗^p ≤ 0.0001. (C) Activation of Scaf neurons does not alter the feeding preference when given a choice between sucrose and water (n = 4). (D) Enhanced feeding caused by activation of Scaf neurons is directed toward nutritious sugars when given a choice between D- and L-glucose (n = 3–4). (E and F) Overexpression of *scaf* does not alter the feeding preference in flies. Scaf was overexpressed using R50H04-GAL4 in sated flies. Preference was tested at 25°C under darkness for 2 hr. (E) Overexpression of Scaf does not alter the preference of feeding when given a choice between sucrose and water (n = 3–4). (F) Enhanced feeding caused by overexpression of *scaf* is directed toward nutritious sugars when given a choice between D- and L-glucose (n = 5–6). Bar graphs and line graphs represent the mean. Error bars represent SEM.

To further test whether activation of Scaf neurons or overexpression of *scaf* enhances feeding by changing the hunger state of the fly, we performed a proboscis extension reflex (PER) assay (Inagaki et al., 2012), as starvation increases the fraction of flies exhibiting a PER. Neither overexpression of *scaf* (Figure 6A) nor activation of Scaf neurons (Figure 6B) resulted in a change in the PER in sated flies. This suggests that neither overexpression of *scaf* nor activation of Scaf neurons induces hunger. Taken together, our data suggest that enhanced feeding motivation caused by *scaf* is dependent on the presence of sugar in food and is independent of the state of the fly.

Scaf neurons may evaluate the nutritional content of the food and may motivate the flies to feed when activated due to the presence of sugar in the food. If this were the case, then the fly would evaluate every substance as nutritious upon artificial activation of Scaf neurons or overexpression of *scaf*. Consequently, the fly would lose its preference for nutritious food. To test this, we performed a two-choice assay in sated flies, giving them a choice between 100 mM sucrose and water. Flies in which Scaf neurons were activated as well as those in which *scaf* was overexpressed preferred sucrose over water (Figures 6C and 6E). As sucrose and water are different in terms of caloric value as well as sweetness, we tested the preference of sated flies for D- or L-glucose, which only differ in caloric value. Both upon overexpression of *scaf* and upon activation of Scaf neurons, sated flies chose D-glucose over L-glucose (Figures 6D and 6F). The data show that the enhanced feeding caused by overexpression of *scaf* or activation of Scaf neurons does not abolish the preference of flies to feed on nutritious sugars. Therefore, Scaf neurons do not evaluate the nutritional content of food.

We also tested whether silencing of Scaf neurons or knockdown of *scaf* results in attenuation of feeding preference in starved flies using the same conditions (data not shown). Contrary to our expectations, we did not see any attenuation of feeding preference (for sucrose versus water or D-glucose versus L-glucose) in the starvation conditions (16 hr) used for testing. This long starvation period may activate other homeostatic mechanisms. Additional starvation conditions need to be tested to verify whether silencing of Scaf neurons or knockdown of *scaf* can attenuate feeding preference.

## Discussion

Recent studies have shown that nutrient balance is a major determinant of behavior. A study in orb-weaving spiders has shown that the nutrient balance of a predator can alter foraging behavior (Mayntz et al., 2009), while in *Drosophila*, intake of macronutrients (particularly carbohydrates) can influence male pre- and post-copulatory reproductive traits (Morimoto and Wigby, 2016). Furthermore, the dietary yeast and sucrose content of the diet has sex-dependent effects on the sleep architecture of the fly (Catterson et al., 2010). In this study, we have determined on a systems level the transcriptional response of the brain to deprivation of a macronutrient, namely carbohydrates. Our data demonstrate that the brain mounts a distinct transcriptional response under these conditions. This distinct response can start to explain the changes in behavior observed upon alterations of individual macronutrients in the diet. Our data also provide us with a repertoire of genes that change expression upon carbohydrate deprivation. This valuable resource can be mined to understand and link molecular mechanisms with specific responses of the brain to carbohydrate deficiency.

Our findings suggest that SPs and SPHs play an important role in modulating fly behavior when the fly is deprived of sugar. We show that the SPH *scaf* positively regulates feeding, depending on the presence of sugar in the food. However, the mechanism of action of *scaf* is not clear. It is possible that Scaf is cleaved into smaller peptides that play a role in neuronal communication or that Scaf competes with an active SP for specific substrates. In embryos, *scaf* expression is upregulated by activation of the JNK pathway and acts as an antagonist of JNK signaling. Hence, Scaf regulates its own expression levels (Rousset et al., 2010). This negative-feedback loop may provide an interesting mechanism to control *ad libitum* feeding in flies. As sugar positively regulates the expression levels of *scaf*, sugar-rich food would induce constitutively high levels of *scaf* expression, which in turn would cause continuous feeding. The autoregulatory capacity of *scaf* may explain the fact that this does not happen in natural conditions, as Scaf downregulates its own expression. Interestingly, pharmacological inhibition of JNK signaling reduces food intake and protects against obesity in diet-induced obese mice (Gao et al., 2017).

Several studies have demonstrated that the brain can detect differences in the caloric content of the available food (Burke and Waddell, 2011, Dus et al., 2011, Dus et al., 2015, Stafford et al., 2012). Our data show that *scaf* expression increases when flies are fed on sugar-rich food. Therefore, Scaf neurons must receive information about the sugar content of the food and respond by regulating the levels of *scaf*. Scaf neurons are located in the SEZ of the adult brain and the VNC, and we currently cannot determine if the effect on feeding is caused only by SEZ neurons. Scaf neurons appear to be second-order neurons and their polarity suggests that they can convey information to higher brain centers. Gustatory neurons from external mouthparts and the pharynx project into the SEZ (Thorne et al., 2004), and the SEZ plays an important role in processing gustatory information (Harris et al., 2015). The dendritic projections of SEZ Scaf neurons around the foramen and in the SEZ therefore indicate that these neurons may be a part of the neuronal circuitry that relays gustatory information to higher brain centers. Similar neurons that transmit information about sugar have been reported earlier (Kim et al., 2017). Scaf neurons could be a parallel set of neurons that transmit information about the sugar content of the food when the fly eats. Scaf neuron activity would motivate the fly to continue feeding on food that is rich in sugars rather than feeding on sugar-deficient food sources. This may be important for survival, as it prevents the fly from feeding on nonnutritious food and encourages the fly to build up energy reserves even when it is no longer hungry.

Regulating food intake is an important process toward the maintenance of energy homeostasis. Neuronal and hormonal mechanisms regulate the feeding drive, depending on the internal state of the body and the quality of the available food (Itskov and Ribeiro, 2013, Saper et al., 2002). The drive to consume palatable, energy-dense food may ensure survival in times of scarcity but when dysregulated may result in overfeeding and obesity (Saper et al., 2002, Yu et al., 2015). Studies in mice suggest that the neural circuits responsible for the homeostatic control of feeding are dispensable when feeding is assessed on a high-fat, high-sugar diet (Denis et al., 2015), thus demonstrating independent homeostatic and hedonic control of feeding. We have shown that *scaf* and Scaf neurons promote feeding on nutritious sugars independent of the hunger state of the fly. Scaf responds to the presence of nutritive sugars in food, and Scaf neurons do not evaluate the quality of food. The enhanced feeding motivation that we notice upon activation of Scaf neurons and upon *scaf* overexpression may be due to its effect on downstream neurons. We feel that manipulation of *scaf* or Scaf neuron activity results in a change in feeding only in sated state due to the fine balance between the internal state of the body and the quality of the food in regulating feeding drive. In the sated state, when the feeding drive due to the internal state is low or absent, increased activity of Scaf neurons or overexpression of *scaf* can easily enhance the feeding drive on nutritive sugars, while silencing Scaf neurons or downregulating the levels of *scaf* reduces the feeding drive. These effects may be due to enhanced or decreased activation of the downstream feeding machinery to which Scaf neurons convey the information about the nutrient content of the food. In starved state, the drive to feed is already high. As pointed out earlier, other circuits also transmit information about sugar content to higher brain centers. The enhanced feeding drive in the starved state coupled with information about the food from other neurons is likely sufficient to drive feeding to an extent that would render the feeding enhancement caused by manipulation of *scaf* or Scaf neurons unobservable.

## STAR★Methods

### Key Resources Table

**Table:** 

REAGENT or RESOURCE	SOURCE	IDENTIFIER
**Antibodies**		

Rabbit anti-Scaf	Stephane Noselli (Rousset et al., 2010)	N/A
Rabbit anti-GFP	Invitrogen	Cat # A11122; RRID: AB_221569
Chicken anti-GFP	Invitrogen	Cat # A10262; RRID: AB_2534023
Rabbit anti-dsRed	Takara	Cat # 632496; RRID: AB_10013483
Goat anti-Chicken IgG (H+L), secondary antibody, alexa fluor 488	Invitrogen	Cat # A-11039; RRID: AB_142924
Donkey anti-mouse IgG (H+L), secondary antibody, alexa fluor 546	Invitrogen	Cat # A-10036; RRID: AB_2534012
Goat anti-Rabbit IgG (H+L), secondary antibody, alexa fluor 488	Invitrogen	Cat # A- 11008; RRID: AB_143165

**Chemicals, Peptides, and Recombinant Proteins**		

TRIzol	Invitrogen	Cat # 15596026
Trehalase from porcine kidney	Sigma	Cat # T8778
Brilliant Blue FCF	Wako Chemicals	Cat # 02712842
Graduated microcapillary tube	Sigma	Cat # P0549
Light Cycler 480 Probes Master	Roche	Cat # 04887301001
Chloroform	Sigma	Cat # 288306
2-propanol	Sigma	Cat # I9516
Triton X-100	Sigma	Cat # T8787
Vectashield mounting medium	Vector Labs	Cat # H-1000
20% Paraformaldehyde (formaldehyde) aqueous solution	Electron Microscopy Sciences	Cat # 15713
Sulforhodamine	Sigma	Cat # S1402
Indigo Carmine	VWR	Cat # 22537.138

**Critical Commercial Assays**		

RNeasy Mini Kit	QIAGEN	Cat # 74104
Glucose (HK) kit	Sigma	Cat # GAHK20-1KT
Superscript III first strand synthesis system	Thermo Fisher	Cat # 18080051

**Deposited Data**		

RNA Sequencing reads	This paper	GEO: GSE107258

**Experimental Models: Organisms/Strains**		

*D. melanogaster*, UAS-*scaf*	Stephane Noselli (Rousset et al., 2010)	N/A
*D. melanogaster,* Scaf protein trap line	DGRC Stock center, Kyoto Institute of Technology	DGRC stock # 109979
*D. melanogaster,* R50H04-GAL4	Bloomington Stock Center	Bloomington stock #46004
*D. melanogaster,* UAS-*scaf*-RNAi	Bloomington Stock Center	Bloomington stock # 55695
*D. melanogaster,* n-*syb*-GAL4	Bloomington Stock Center	Bloomington stock # 51635
*D. melanogaster,* UAS-mCD8::GFP	Bloomington Stock Center	Bloomington stock # 5137
*D. melanogaster,* UAS-DenMark-RFP	Nicolaï et al., 2010	N/A
*D. melanogaster,* UAS-GFP-Syd-1	Owald et al., 2010	N/A
*D. melanogaster,* UAS-dTrpA1	Hamada et al., 2008	N/A
*D. melanogaster,* UAS-TNT	Sweeney et al., 1995	N/A
*D. melanogaster, tub*P-GAL80^ts^	McGuire et al., 2003	N/A

**Oligonucleotides**		

Primers for qRT-PCR	This paper	Table S3

**Software and Algorithms**		

Trimmomatic	Bolger et al., 2014	http://www.usadellab.org/cms/?page=trimmomatic
Tophat2	Kim et al., 2013	https://ccb.jhu.edu/software/tophat/index.shtml
Cufflinks	Trapnell et al., 2012	http://cole-trapnell-lab.github.io/cufflinks/

### Contact for Reagent and Resource Sharing

Further information and requests for resources and reagents should be directed to and will be fulfilled by the Lead Contact, Korneel Hens (Korneel.hens@cncb.ox.ac.uk).

### Experimental Model and Subject Details

The fly strains that have been used in this study are UAS-mCD8::GFP, UAS-mCD8 chRFP, UAS-DenMark-RFP (Nicolaï et al., 2010), UAS-GFP-Syd-1 (Owald et al., 2010), UAS-dTrpA1 (Hamada et al., 2008), UAS-TNT (Sweeney et al., 1995), *tub*P-GAL80^ts^ (McGuire et al., 2003), UAS-*scaf* (Rousset et al., 2010). The Scaf protein trap line was obtained from the KYOTO Stock Center (DGRC stock # 109979) in the Kyoto Institute of Technology. R50H04-GAL4 (stock #46004) (Jenett et al., 2012), *scaf*-RNAi (stock #55695) and n-*syb*-GAL4 (stock #51635) were obtained from the Bloomington stock center.

### Method Details

#### Fly strains and rearing

Flies were maintained on cornmeal/agar under 12 hour light / 12 hour dark cycle at 25°C unless mentioned otherwise. For GAL80 experiments, flies were reared at 18°C until eclosion. For TrpA1 experiments, flies were reared at 21°C until eclosion. For all behavioral experiments, 4 to 5 day old mated male flies were separated from female flies so that feeding quantification could be carried out in a sex-specific manner. The male flies were placed in groups of 10 to 12 flies each and were allowed to recover for at least 48 hours before carrying out behavioral experiments. For RNA isolation and trehalose measurements, wandering third instar larvae were removed from food bottles, washed in Phosphate-Buffered Saline (PBS) pH 7.4 and transferred to holidic food. Flies that emerged were maintained on holidic medium for 5 days post eclosion and subsequently starved for 24 hours on holidic medium lacking sugar (sugar starvation), on 2% agar (complete starvation) or on complete holidic medium (controls). Male flies were used for dissection and subsequent RNA isolation.

#### Trehalose measurement

Haemolymph of adult flies was collected by piercing the thorax of adult flies using insect pins (0.1 mm). Fifty adult male flies were placed in a 0.5 mL microfuge tubes with a small hole in the bottom, which was placed in 1.5 mL microfuge tube. This microfuge tube was centrifuged at 1500 g for 5 min at 4°C. Around 1μL of clear haemolymph was collected, diluted using trehalose buffer (5mM Tris, pH 6.6, 137 mM NaCl, 2.7mM KCl) and digested using Porcine Trehalase (Sigma) at 37°C for 18 hours. The glucose (HK) assay kit (Sigma) was subsequently used to measure the levels of free glucose post digestion. Absorbance of the digested mix was measured at 340 nm using a 96-well plate reader (BMG Labtech) and glucose concentrations were calculated using a glucose standard curve.

#### RNA isolation and high-throughput sequencing

RNA was isolated from dissected brain tissue from four independent biological replicates of 120-130 brains each using TRIzol (Invitrogen) and the QIAGEN RNeasy mini kit according to manufacturer’s guidelines. The quality of RNA isolated was checked using a Bioanalyzer (Agilent) and sequenced with polyA selection on an Illumina HiSeq 2500 system, generating 100bp paired-end reads. Raw data has been submitted to the Gene Expression Omnibus (GEO) database (Accession number GSE107258). Raw reads were trimmed using Trimmomatic (Bolger et al., 2014) to remove the adaptor sequences and low quality reads. The reads were aligned to the *Drosophila* genome version dm3 using Tophat2 (Kim et al., 2013) and transcripts were assembled using Cufflinks and Cuffmerge. Subsequently, differentially expressed transcripts were identified using Cuffdiff. We applied a log2 fold change cut-off of 0.5 for the complete starvation dataset and a cut-off of 0.3 for sugar starvation dataset to pick up genes for further analysis.

#### qRT-PCR

cDNA was generated from 500 ng of total RNA using Superscript reverse transcriptase III (Thermo Fisher) according to manufacturer’s guidelines. Primer design and probe selection was done in the Roche assay design center. Primer sequences are described in Table S3. qRT-PCR was performed on a Roche LightCycler 480 machine.

#### GO analysis and representation

Genes were classified into different ontological categories using DAVID (https://david.ncifcrf.gov). Enrichment of GO categories in the two datasets were compared using the enrichment map plugin (http://www.baderlab.org/Software/EnrichmentMap) in Cytoscape. The representation factor and its corresponding hypergeometric probability was calculated on http://nemates.org/MA/progs/overlap_stats.html. The representation factor is the number of overlapping genes divided by the expected number of overlapping genes drawn from two independent groups.

#### Immunostaining

Brains were dissected in ice cold PBS and fixed in 4% formaldehyde at room temperature for 25 min. Immunostaining was carried out on fixed brains as described earlier (Wu and Luo, 2006). Briefly, the dissected brains were washed after fixation using PBS containing 0.3% Triton X-100 (referred to as PBTX). Blocking was carried out using 5% normal goat serum (NGS) in PBTX for 30 min at room temperature. The brains were incubated for 48 hours at 4°C in primary antibody diluted in PBTX. Following incubation, the brains were washed thoroughly using PBTX at room temperature (4 washes of 10 min each) and incubated in secondary antibody for another 48 hours at 4°C. The brains were washed again with PBTX at room temperature and mounted using Vectashield mounting medium (Vector Labs). Primary antibodies used in this study are rabbit anti-Scaf (1:200), a kind gift from Stephane Noselli, rabbit anti-GFP (1:500) (Invitrogen), chicken anti-GFP (1:200) (Invitrogen), rabbit anti-dsRed (1:500) (Takara). Secondary antibodies used in this study are fluorophore conjugated (Alexa 488/ 546) anti-mouse (1:500), anti-chicken (1:200) or anti-rabbit (1:500) (Invitrogen).

#### Feeding assays

For dye-based assays, brilliant blue FCF (0.4%) (Wako Chemicals) was mixed with a 100mM sucrose solution containing 1% agar. For testing the specificity of feeding toward fructose, a similar assay was performed using 100mM fructose. An even layer of this mix was spread on a filter paper, which was subsequently rolled on the inner walls of the fly tube. Flies were gently tapped into the tube and incubated at the required temperature. Post feeding, flies were quickly transferred into a plastic tube and frozen by placing the tubes at −20°C. Male flies were then crushed in PBS (20 μl/ fly), centrifuged at 10000 g for 5 min and the supernatant was removed. Absorbance of the supernatant was measured at 625 nm using a 96-well plate reader. The amount of food eaten was represented in terms of absorbance units, which we have referred to as arbitrary units.

For the CAFÉ assays, fly vial plugs were prepared with plastic adaptors using pipette tips that can hold capillary tubes. Water was supplied as 1% agar on the bottom of the fly vials. 100mM sucrose solution or 100 mM fructose solution was provided in graduated capillary tubes (Sigma). To prevent evaporation, the top of the capillary tube was sealed with mineral oil. We also used evaporation control vials without flies. Net feeding was quantified by subtracting the amount of solution lost due to evaporation from total sucrose consumption. Feeding per fly was calculated by dividing the net feeding in each vial by the number of flies in individual vials and is represented in terms of volume of sucrose or fructose fed per fly. P values were calculated using unpaired t tests using the software Prism.

#### Survival assay

Seven to eight day old adult flies were kept on starvation medium (1% agar in water) at 25°C. The number of dead flies was counted in regular intervals and survival was represented as the percent of flies surviving after a particular interval.

#### PER assay

Flies were briefly anaesthetized on ice and stuck on their backs using nail varnish on a glass slide. The flies were kept in a moist chamber and allowed to recover for at least 40 min. Prior to testing, water was provided using a pipette tip until the flies stopped drinking to ensure they were not thirsty prior to testing. Subsequently, increasing concentrations of sucrose solution were presented to the flies by soaking a cotton bud in sucrose solution and the extension of the proboscis was scored. Each fly was presented thrice with the soaked cotton bud and an extension one out of three times was counted as a positive response. PER was carried out in groups of 10 flies and the number of flies in each group that showed a positive response was recorded. The average number of positive responders among different groups was used to represent the fraction of responders to individual sucrose concentrations.

#### Two choice assay

Solutions of 100mM sucrose, D- or L- glucose were prepared and mixed with Indigo carmine (VWR) or sulforhodamine (Sigma) to impart blue or red color to the solutions respectively. The final concentration of these chemicals was 0.25 mg/mL. The solutions were mixed in 1% agar and spotted on a Petri plate in a symmetrical array. Flies were transferred to the Petri dishes and allowed to feed for two hours in the dark at the desired temperature. After 2 hours, the Petri plates containing the flies were frozen and the number of flies with colored abdomens were counted. The preference index for substance T (PI^T^) was calculated as: PI^T^ = (N^T^ + N^M^/2)/(N^T^+ N^C^+ N^M^) where N^T^ is the number of flies that fed on substance T, N^C^ is the number of flies that fed on substance C (the other substance) and N^M^ is the number of flies that fed both on substance T and C.

### Quantification and Statistical Analysis

Statistical analysis was carried out using Prism software. Unpaired t- test was used to test the differences between different groups of flies. The number of groups of flies (n) used for individual assays has been described in the legend of each figure. For qRT- PCR experiments (n) represents technical replicates. Error bars in qRT-PCR graphs represent standard deviation (SD), all other error bars represent standard error of the mean (SEM). Whiskers in all boxplots go from the minimum to the maximum value while the box extends from 25th to 75th percentile. All values of individual groups of flies are shown as dots and the line in the box is plotted at the median. Bar graphs and line graphs represent the mean.

^∗^p ≤ 0,05; ^∗∗^p ≤ 0.01; ^∗∗∗^p ≤ 0.001; ^∗∗∗∗^p ≤ 0.0001

### Data and Software Availability

The accession number for the raw RNA-seq data reported in this paper is GEO: GSE107258.

## Supplemental Information

Document S1. Figures S1–S6 and Table S3

Table S1. Genes Differentially Expressed under Complete Starvation Regime, Related to Figure 1

Table S2. Genes Differentially Expressed under Sugar Starvation Conditions, Related to Figure 1

Data S1. Cytoscape Network of GO Term Enrichment Associated with Sugar-Absence and Macronutrient-Presence Datasets, Related to Figure 2 Comparison of the GO term enrichment associated with Sugar-Absence (SA) and Macronutrient-Presence (MP) datasets visualized by the Cytoscape enrichment app. The node (inner circle) size corresponds to the number of genes in the MP dataset while the node border (outer circle) size corresponds those in SA dataset within the given biological process. The color of the node and border corresponds to the significance of the enrichment. Edge size corresponds to the number of genes that overlap between the two connected biological processes. Green edges correspond to the MP dataset and blue corresponds to SA dataset.

Document S2. Article plus Supplemental Information
