# Supplementary material for: Sugar Promotes Feeding in Flies via the Serine Protease Homolog scarface
Source: Cell Rep. 2018 Sep 18;24(12):3194–3206.e4. doi: 10.1016/j.celrep.2018.08.059 (PMC6167639; doi:10.1016/j.celrep.2018.08.059)
Supplement: Document S1. Figures S1–S6 and Table S3 [file mmc1.pdf]

Cell Reports, Volume *24*

Supplemental Information

Sugar Promotes Feeding in Flies via the Serine Protease Homolog *scarface*

Naveen Prasad and Korneel Hens

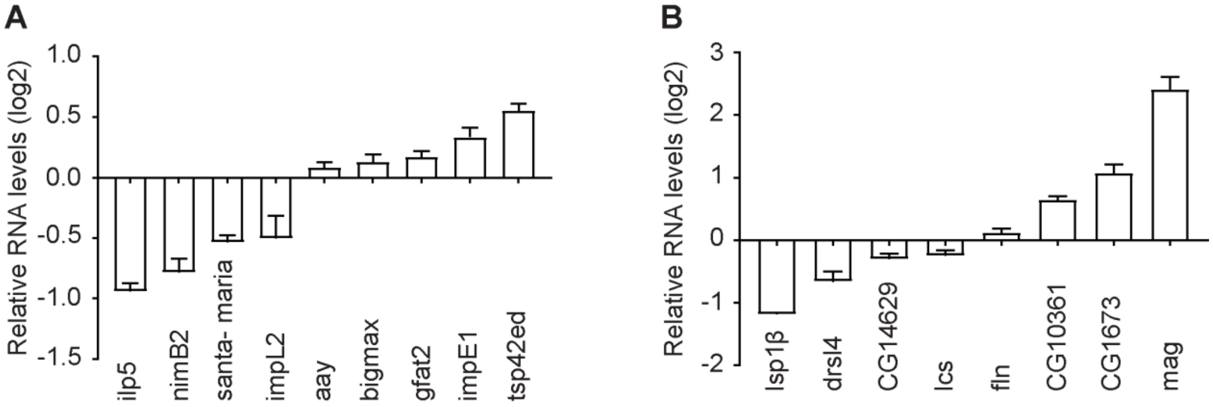

Figure S1: Validation of RNA-seq data. Related to Figure 1.

(A-B) Changes in transcript levels of different genes as measured by qRT-PCR on an independent biological replicate under conditions of complete starvation (A) and sugar starvation (B). N = 3 (technical replicates).

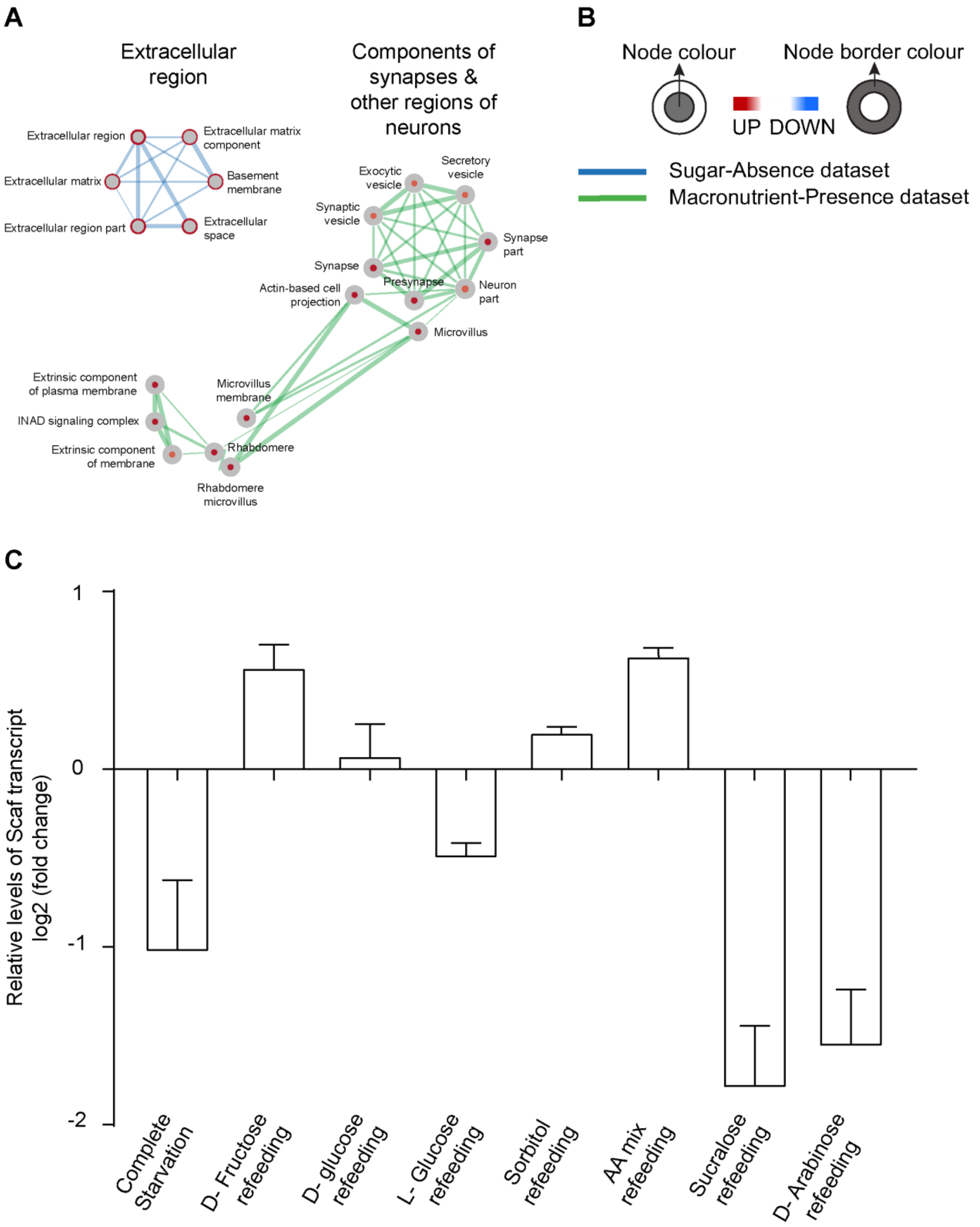

Figure S2: Genes responding to the absence of sugar encode mainly secreted proteins. Related to Figure 2.

(A) Enrichment of the cellular localisation of genes associated with Sugar-Absence (SA) and Macronutrient-Presence (MP) datasets visualised by the Cytoscape enrichment app. The node (inner circle) size corresponds to the number of genes in the MP dataset while the node border (outer circle) size corresponds to those in the SA dataset within the given biological process. The colour of the node and border corresponds to the significance of the enrichment. Edge size (lines connecting the nodes) corresponds to the number of genes that overlap between the two connected biological processes. Green edges correspond to the MP dataset and blue corresponds to SA dataset. The network map was

manually curated removing general and uninformative sub-networks. The full network map can be seen in Network file S1.

(B) Legend for panel A

(C) Relative transcript levels of *scaf* in heads of flies under different feeding conditions. Flies were starved of all nutrients for 24 hours on 1% agar and subsequently starved for an additional 3 hours (complete starvation) or fed with 200mM of different sugars (as indicated) or with a mix of amino acids (AA mix) in 1% agar for 3 hours. The concentration of the AA mix was equivalent to that used for the preparation of the holidic medium. Scaf expression levels in fly heads were measured by qRT-PCR and compared to *scaf* expression levels of flies that were fully fed for 27 hours (controls). N = 3 (technical replicates).

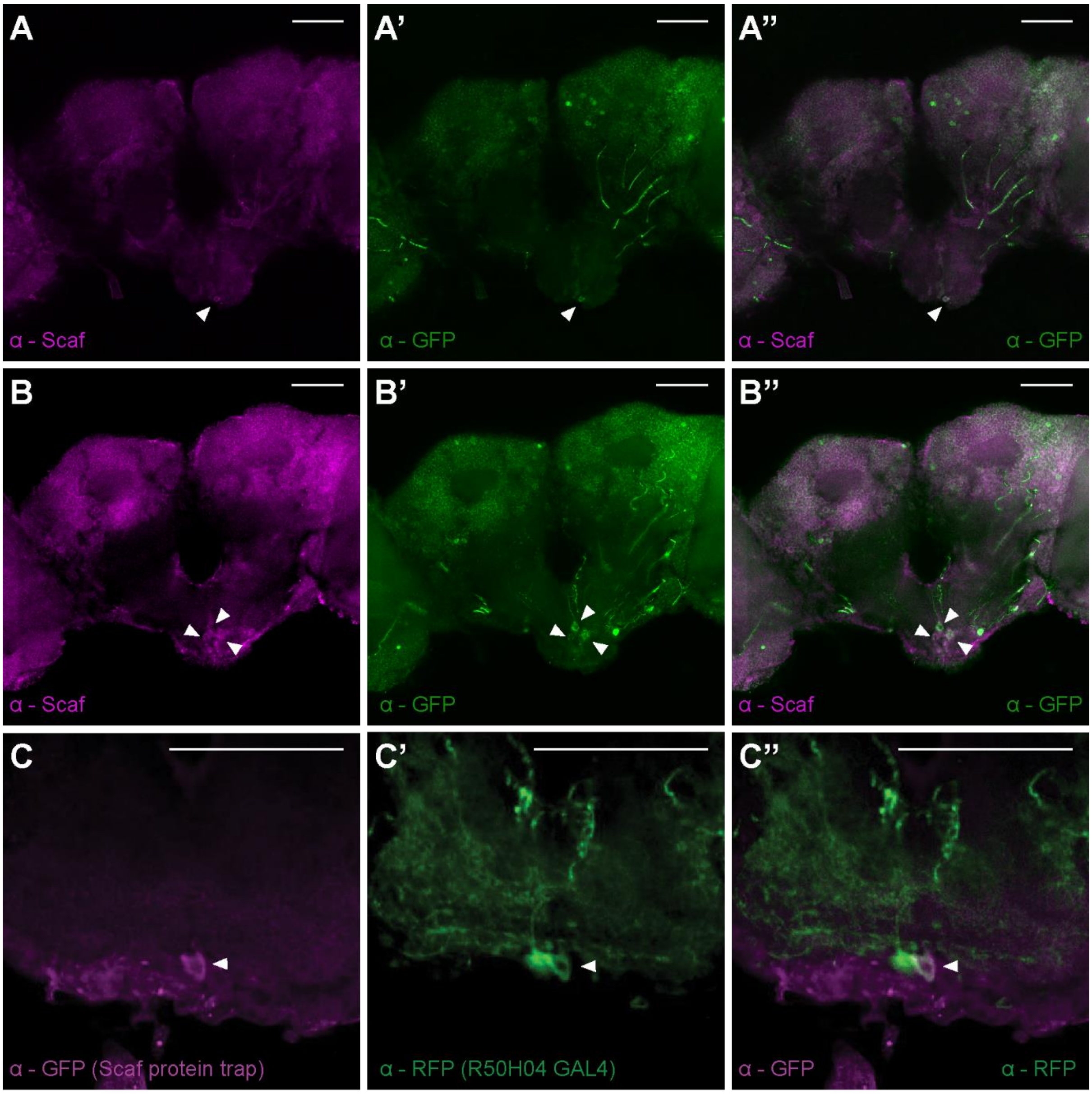

Figure S3: Scaf is expressed in neurons labeled by R50H04 GAL4 line. Related to Figure 3.

(A-B'') Adult brains from R50H04-GAL4; UAS-6XGFP flies double stained using anti-GFP (green) and anti-Scaf (magenta) antibodies demonstrating that Scaf is expressed in R50H04 neurons. Arrowheads mark cell bodies expressing Scaf and GFP. A-A'' and B- B'' show the expression of Scaf and GFP in two different focal planes.

(C-C'') Adult fly brain from R50H04-GAL4/$scaf^{KM0624}$;UAS-mCD8::Cherry co-stained with anti-RFP (green) and anti-GFP (magenta) demonstrating that endogenously labeled Scaf is expressed in neurons labeled by the R50H04-GAL4 driver.

Scale bar: 50 µm

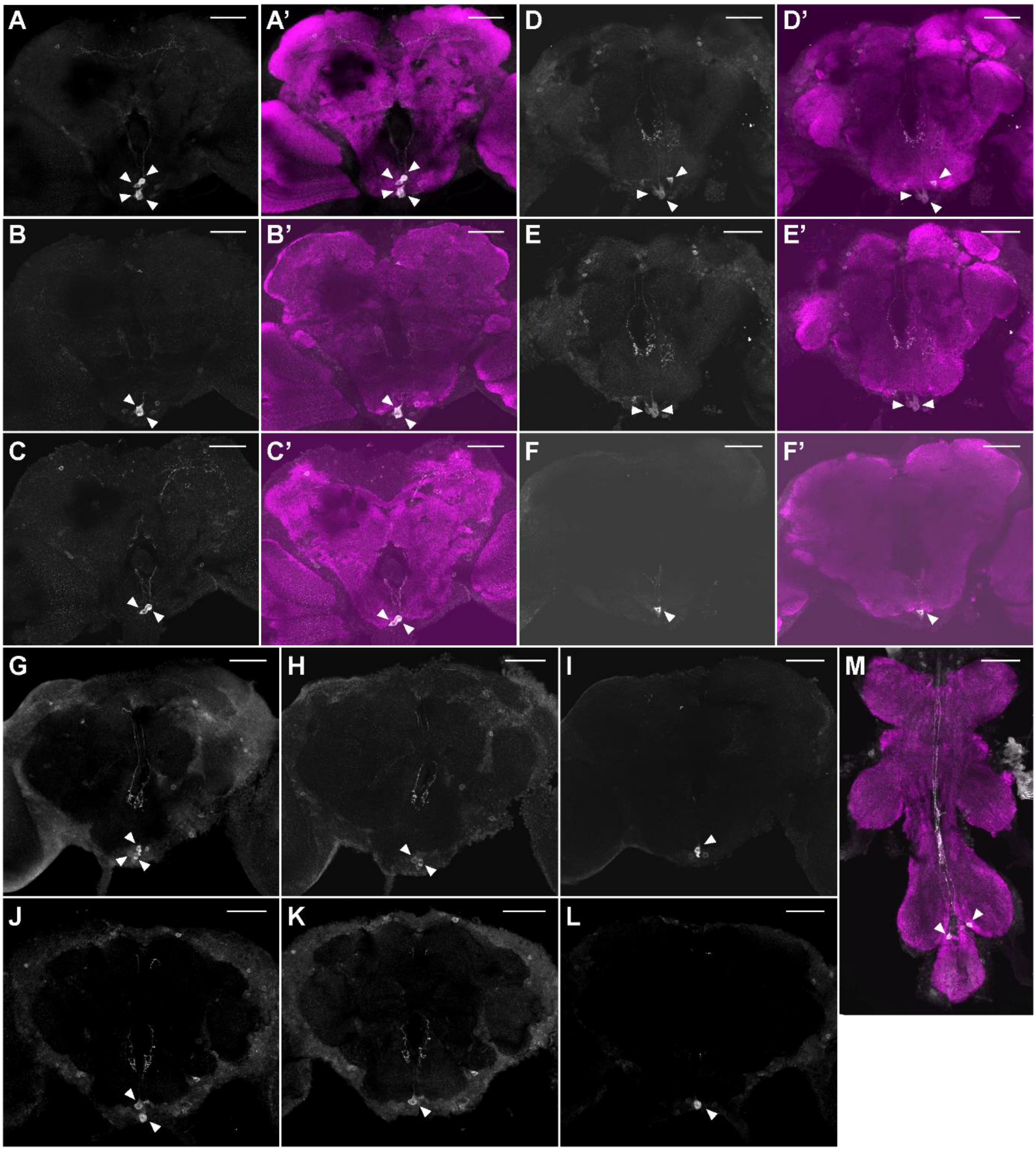

Figure S4: Cell bodies of Scaf neurons are present in the SEZ of the brain and in the ventral nerve cord (VNC). Related to Figure 3.

(A-L) Images of four different brains as Z projections of the entire brain or an anterior or posterior substack.

(A-C') Z projection of a brain from R50H04; UAS mCD8::GFP flies stained for anti-GFP (white) with (A', B' C') or without (A, B, C) anti-nc82 (magenta) counterstaining. (A, A') Z projection images from the entire brain. (B, B') Z Projections from an anterior subset of the confocal stack. (C, C') Z Projections from a posterior subset of the confocal stack.

(D-F') Same as (A-C') for a second brain.

Please note that at lower laser intensities (A, A') only the cell bodies in SEZ region of brain are clearly visible. The neurites, especially the ones present in the anterior region and around the oesophagus are visible but faint. At higher

laser intensities, the neurites around the oesophagus are more clearly visible but background staining starts to light up. As nc82 staining in our hands is very bright in the posterior part of brain, the anterior antennal lobes are not clear in the Z- projection (A', D'). Full stacks of the images can be provided upon request.

(G-I) Z projection of a third brain from R50H04; UAS mCD8::GFP flies stained for anti-GFP (white). (G) Z projection from the entire brain. (H) Projection from an anterior subset of the confocal stack. (I) Z Projection from a posterior subset of the confocal stack.

(J-L) Same as (G-I) for a fourth brain.

(M) Z projection of the VNC from R50H04; UAS mCD8::GFP flies stained for anti-GFP (white) counterstained with nc82 (magenta).

Arrowheads indicate cell bodies of Scaf neurons.

Scale bar: 50 µm

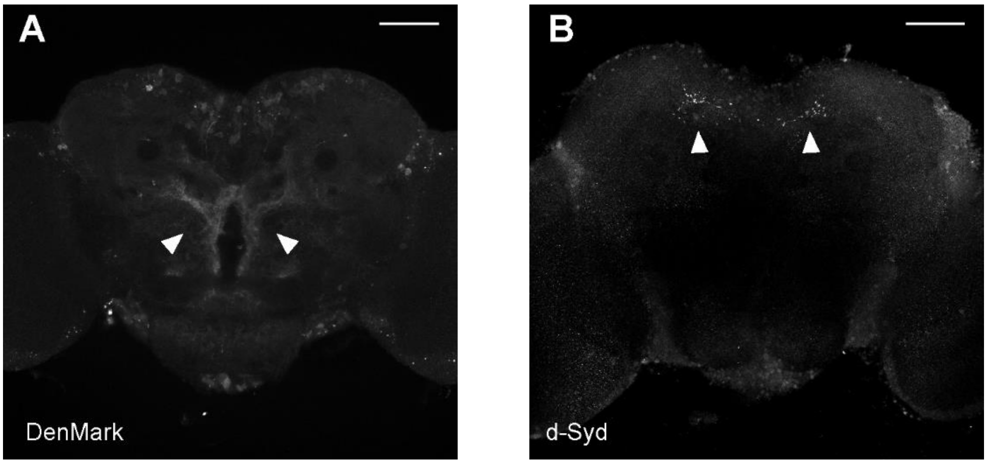

Figure S5: Scaf neurons have dendritic projections around foramen and presynaptic processes in the protocerebrum. Related to Figure 3.

(A) Adult brains from R50H04-GAL4; UAS-DenMark-RFP flies stained using anti-RFP to show the post-synaptic termini of Scaf neurons.

(B) Adult brains from R50H04-GAL4; UAS-GFP-dSyd flies using anti-GFP to show the pre-synaptic termini of Scaf neurons.

Scale bar: 50 µm

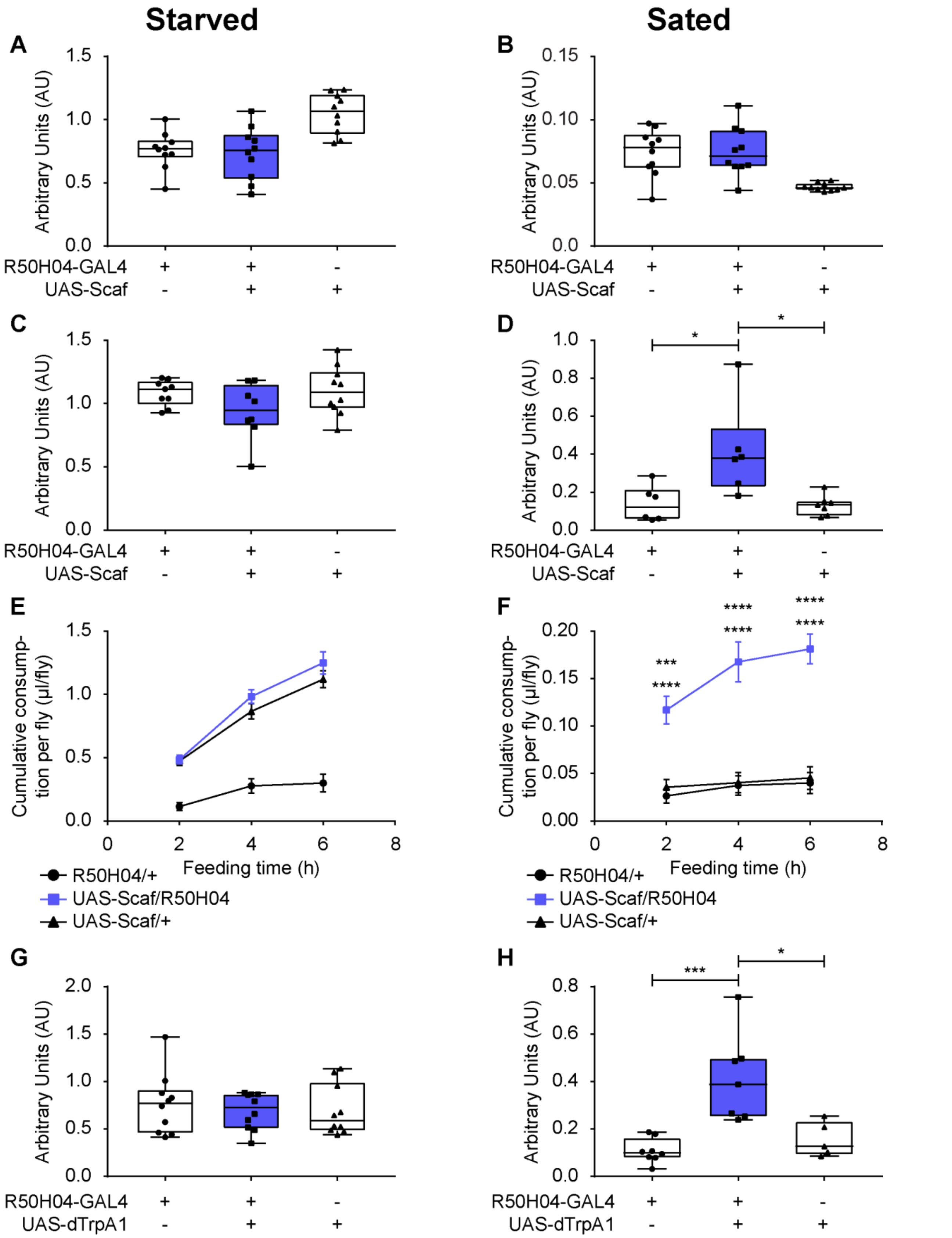

Figure S6: Scaf promotes feeding of fructose in flies. Related to Figure 4 and 5.

(A-B) Effect of *scaf* overexpression on feeding of fructose in flies over a 15-minute period. Scaf overexpression was driven by R50H04-GAL4 and feeding was quantified using the dye assay at 25ºC for 15 minutes. (A) Overexpression of *scaf* does not change starvation-induced feeding in flies upon 16 hours of starvation (n = 10). (B) Overexpression of

scaf does not change feeding in sated flies as compared to the genetic controls in 15 minutes unlike sucrose feeding (n = 10).

(C-D) Effect of *scaf* overexpression on feeding of fructose in flies over a 30-minute period. Scaf overexpression was driven by R50H04-GAL4 and feeding was quantified using the dye assay at 25ºC for 30 minutes. (C) Overexpression of *scaf* does not change starvation-induced feeding in flies upon 16 hours of starvation (n = 8-10). (D) Overexpression of *scaf* results in enhancement of feeding in sated flies as compared to the genetic controls (n = 6-7).

(E-F) Effect of *scaf* overexpression on feeding of fructose over longer periods. Scaf overexpression was driven by R50H04-GAL4 and feeding was quantified at 25ºC for 2, 4 and 6 hours using the CAFÉ assay (E) Overexpression of *scaf* does not change starvation-induced feeding in flies upon 16 hours of starvation (n = 9-10). (F) Overexpression of *scaf* results in enhancement of feeding in sated flies as compared to the genetic controls (n = 9-10).

(G-H) Effect of Scaf neuron activation on feeding of fructose. dTrpA1 expression was driven by R50H04-GAL4 to activate Scaf neurons for 1 hour by shifting the flies to from 21ºC to 31ºC. Feeding was subsequently quantified in flies using the dye assay for 10 minutes at 31ºC. (G) Activation of Scaf neurons does not change post-starvation feeding upon 16 hours of starvation (n = 10). (H) Activation of Scaf neurons results in increased feeding in sated flies as compared to the genetic controls (n = 5-8).

Table S3: Sequences of primers used for q-RT-PCR. Related to STAR Methods.

Primer	Sequence (5'- 3')
ilp5 FP	GCCTTGATGGACATGCTGA
ilp5 RP	TCATAATCGAATAGGCCCAAG
nimB2 FP	GTGTGCTACAAGGAAGTTCCAA
nimB2 RP	CGGATTTCGCTCATAGCC
santa- maria FP	CGAGGAGCCTGTGATTTGTT
santa- maria RP	GCGATGGTGTGTGGATACC
impL2 FP	CGAGTGAACGTCATCCAAAA
impL2 RP	TACCAGGTCCACGGCTCTT
aay FP	CCCCTGAAAAACGTCTATGC
aay RP	AGCTATCGTATTCGCCCAAA
bigmax FP	TGAGAACATGCTACAGCATCAG
bigmax RP	TCCATAATGGCCTGGAACA
gfat2 FP	CAACTTCGCCACCTGTTTG
gfat2 RP	ATTCCTTCGCTGTGCATGT
impE1 FP	CAATGGAAACGCTTTTACGG
impE1 RP	GCGTCCACCACTGTGAAGA
tsp42ed FP	GCGGCGTTTTTGTGAAATA
tsp42ed RP	GCAAAATGCCGCATATCA
lsp1beta FP	ACCTGCCCAAGTACACCAAG
lsp1beta RP	TCCACGTTCTTCATGGTCAC
drsl4 FP	GTGGATTGCCCATCTGGA
drsl4 RP	GACGGCACTGCTCTCCAT
CG14629 FP	TGTGGACCAGCTAAGGTCAA
CG14629 RP	AAGGTCACATTGGTGTTTTGC
lcs FP	GAATCCTCGACGGAATCATC
lcs RP	GGGAACATGTCAAGCGATTT
fln FP	TGCGCACACGGAATATCA
fln RP	GCCAGTGTTTGAATTAGTTGTTTG
CG10361 FP	AACAACTACTTAGGACTGGCGAAC
CG10361 RP	CGCCATACTGCTCCAACA
CG1673 FP	TGGCAATTAACGCAAAGGAT
CG1673 RP	GCCGACGATTTCTGTTGC
mag FP	CAACGCCTTCATAATGTTTGC
mag RP	TCTCGATCAGGACGCTCAT
Rpl 30 FP	ATTGTCAGCCGACGAAGTG
Rpl 30 RP	CTCCAGAGCCTTCTTTTGTTTC
scaf FP	GTCAGTGCCTGAACGGATATT
scaf RP	TGGTTTCCGGATCACAGTTT
